# Postoperative Maxillary Cyst Following Maxillary Tooth Extraction: A Rare Surgical Predicament

**DOI:** 10.7759/cureus.66407

**Published:** 2024-08-07

**Authors:** Shrabasti Dey, Swapan K Majumdar, Asish K Das, Subhasish Burman, Abira Chattopadhyay

**Affiliations:** 1 Oral and Maxillofacial Surgery, Dr. R. Ahmed Dental College and Hospital, Kolkata, IND

**Keywords:** oral and maxillofacial surgery, oral pathology, maxillary sinus lesions, non-odontogenic cyst, implantation cyst, surgical ciliated cyst, postoperative maxillary cyst

## Abstract

A postoperative maxillary cyst (POMC) is an epithelium-lined cyst that can develop following surgery or trauma in the maxillary antral region. This condition arises from the entrapment of the sinonasal mucosa in the maxilla, and rarely in the mandible, due to trauma or instrumentation near the maxillary sinus. Literature indicates that POMCs, or surgical ciliated cysts, can appear as delayed complications from five months to 56 years after trauma or surgical procedures in the sinus area. Despite its potential for aggressive local destruction, it often presents incidentally with minimal symptoms. This clinical case report describes the occurrence of such a cyst in a 30-year-old male and discusses the diagnosis and management of this rare pathology.

## Introduction

First described by Kubo in 1927, the postoperative maxillary cyst (POMC) - also known as a surgical ciliated cyst, respiratory implantation cyst, postoperative paranasal cyst, ectopic ciliated cyst, or postoperative maxillary mucocele - is a true cyst lined with respiratory epithelium [1-6]. While it is frequently reported in Japan, primarily due to the Caldwell-Luc procedure used historically to treat chronic sinusitis before the advent of functional endoscopic sinus surgery (FESS), it is less common elsewhere [1,3]. Basu et al. (1988) identified it as a rare delayed complication of maxillary antral surgery or trauma, with a prevalence of 1.5% among all oral cysts [4,7]. Although Japanese studies suggest it is uncommon among non-Orientals, other research indicates that it may be underdiagnosed in Western countries [7].

POMCs often present with symptoms such as pain, swelling of the cheek or palate, and discomfort in the maxilla or adjacent teeth, commonly in the maxillary molar region. However, in some cases, these cysts may be asymptomatic and found incidentally [1,5]. With the increasing prevalence of surgeries like bimaxillary orthognathic surgery and combined rhinoplasty/genioplasty, these lesions can also occur in the mandible [4-6]. Known for their potential to cause local destruction, they may mimic a locally aggressive cyst or tumor with extensive bone loss, cortical perforation, extension into adjacent structures, or fistula formation [1,2,4]. Histopathologically, they feature a ciliated respiratory epithelial lining within a fibrous wall, which may show inflammation and reactive bone formation [2]. Literature indicates a recurrence rate of 6-20% following treatment by enucleation and curettage [4].

This case report details the occurrence of a POMC following tooth extraction and discusses its etiology, pathogenesis, diagnosis, and management. It aims to raise awareness that even minor surgical procedures, such as traumatic tooth extractions, can lead to cyst formation.

## Case presentation

A 30-year-old male presented with pain and swelling on the left side of his face, persisting for three months. Eight months prior, he had undergone the extraction of a carious upper left first molar. Two months after the extraction, he developed pain and swelling around the extraction site. He visited a private practitioner, who diagnosed him with a cystic lesion. The patient underwent enucleation of the cyst three months post-extraction, leading to the resolution of pain and swelling. Histopathological examination identified the lesion as a nonspecific odontogenic cyst. However, two months later, he experienced a recurrence of pain and swelling in the same area. There was no history of facial trauma or maxillary sinusitis.

**Figure 1:**
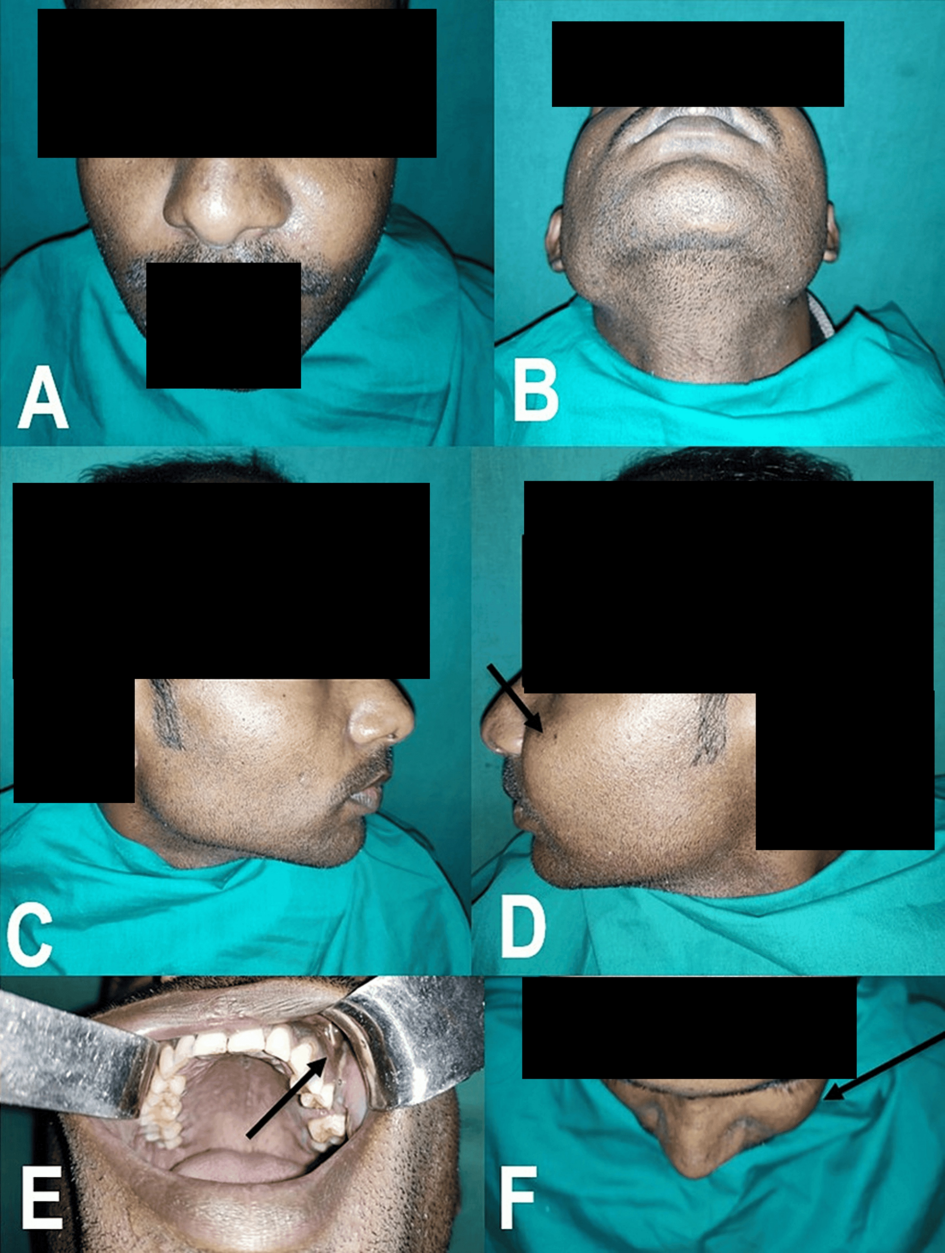
(A-F) Preoperative photographs depicting swelling in the left cheek region

On examination, a solitary, diffuse, tender swelling was observed in the left cheek region. The overlying skin was similar to adjacent areas and was not adherent to the lesion. Intraorally, a discharging sinus was present in the buccal vestibule over the upper left first premolar (Figure 1D). Gingival paraesthesia was noted over the upper left canine and premolars (Figure 1A-1F).

**Figure 2:**
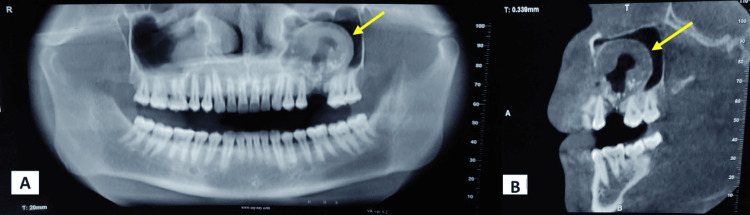
CBCT showing a corticated unilocular radiolucent lesion with a thickened lining and radiopaque flecks, originating from the floor of the left maxillary antrum CBCT, cone beam CT

A cone-beam CT revealed a corticated, unilocular radiolucent lesion with a thickened lining and radiopaque flecks in the left maxillary antrum, originating from its floor (Figure 2A, 2B).

**Figure 3:**
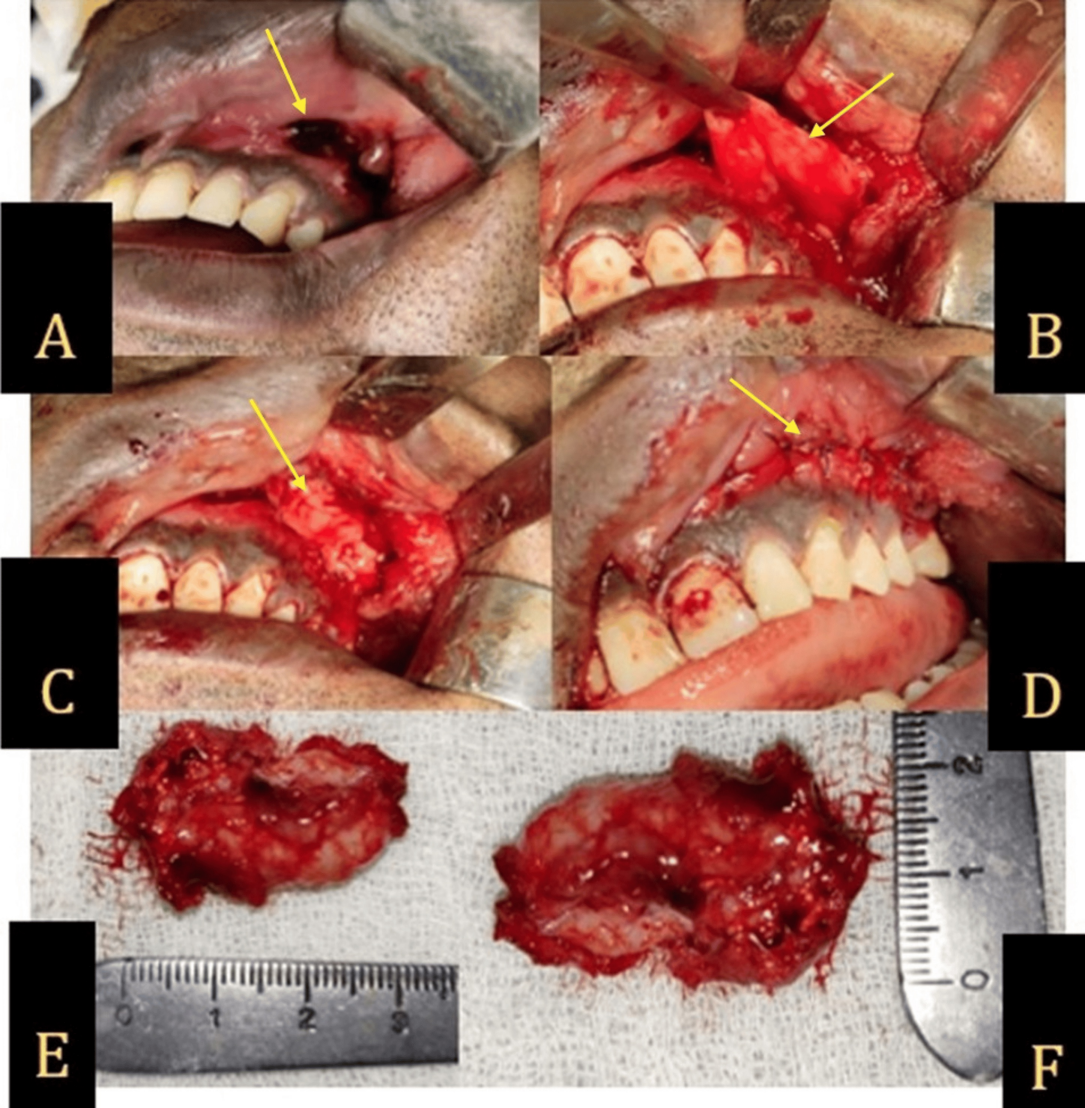
(A) A residual defect following the enucleation of the lesion indicated by the arrow. (B) Buccal fat pad. (C) Buccal fat pad sutured over defect for closure. (D) Second layer of closure with oral mucosa. (E, F) Enucleated lesion, measuring 3 cm × 2 cm

Aspiration of the lesion yielded no fluid. Given the history of previous extractions and attempted enucleation, a residual cyst was initially suspected. However, an incisional biopsy confirmed the diagnosis of a POMC. The patient then underwent complete enucleation of the cyst, including the sinus lining, with double-layered closure using a pedicled buccal fat pad flap and mucosa under general anesthesia (Figure 3A-3F).

**Figure 4:**
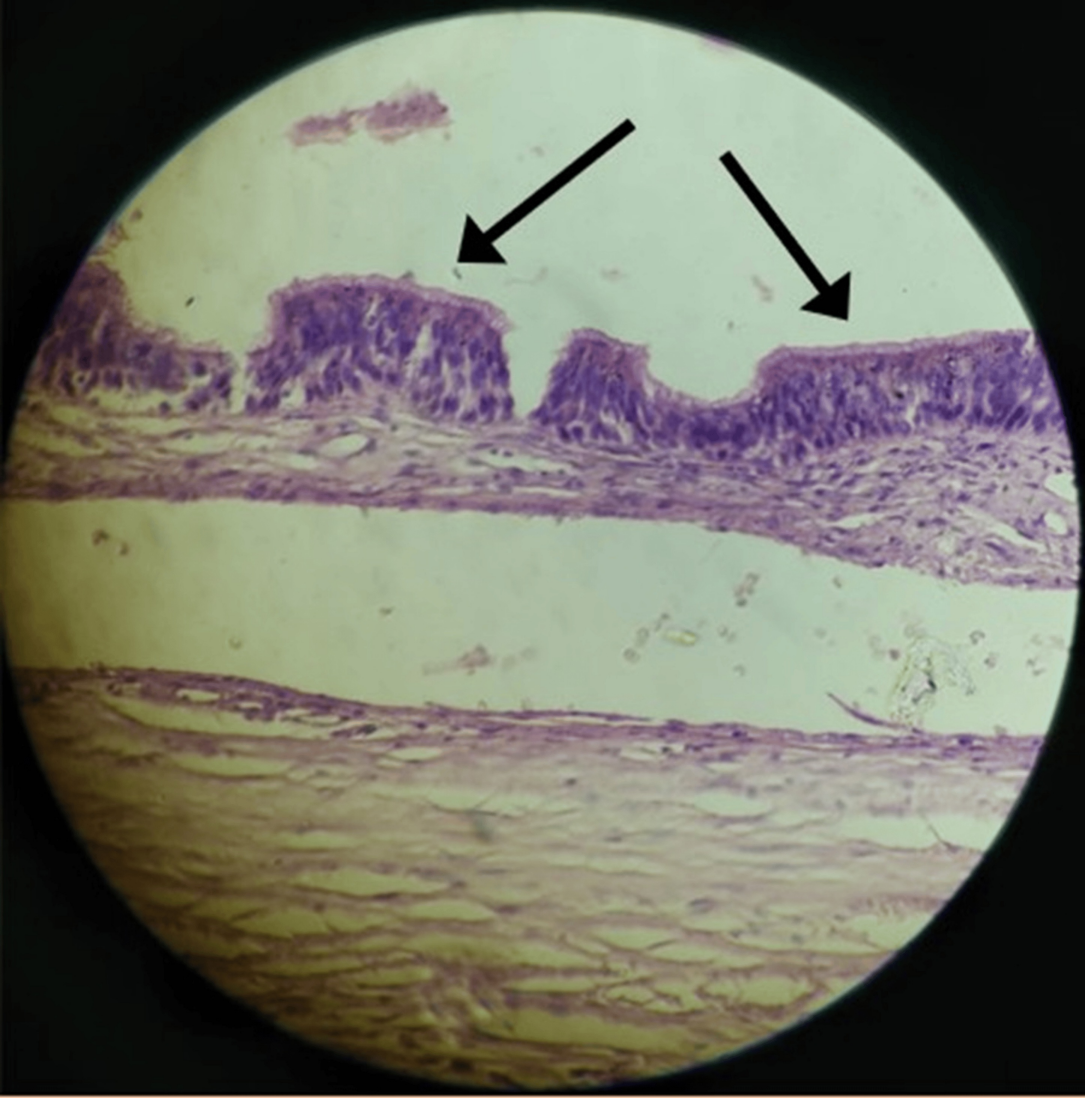
Photomicrograph showing pseudostratified ciliated columnar epithelium with a fibrovascular capsule

Histopathological examination revealed a pseudostratified ciliated columnar epithelial lining with a mature fibrovascular capsule, consistent with a POMC (Figure 4).

**Figure 5:**
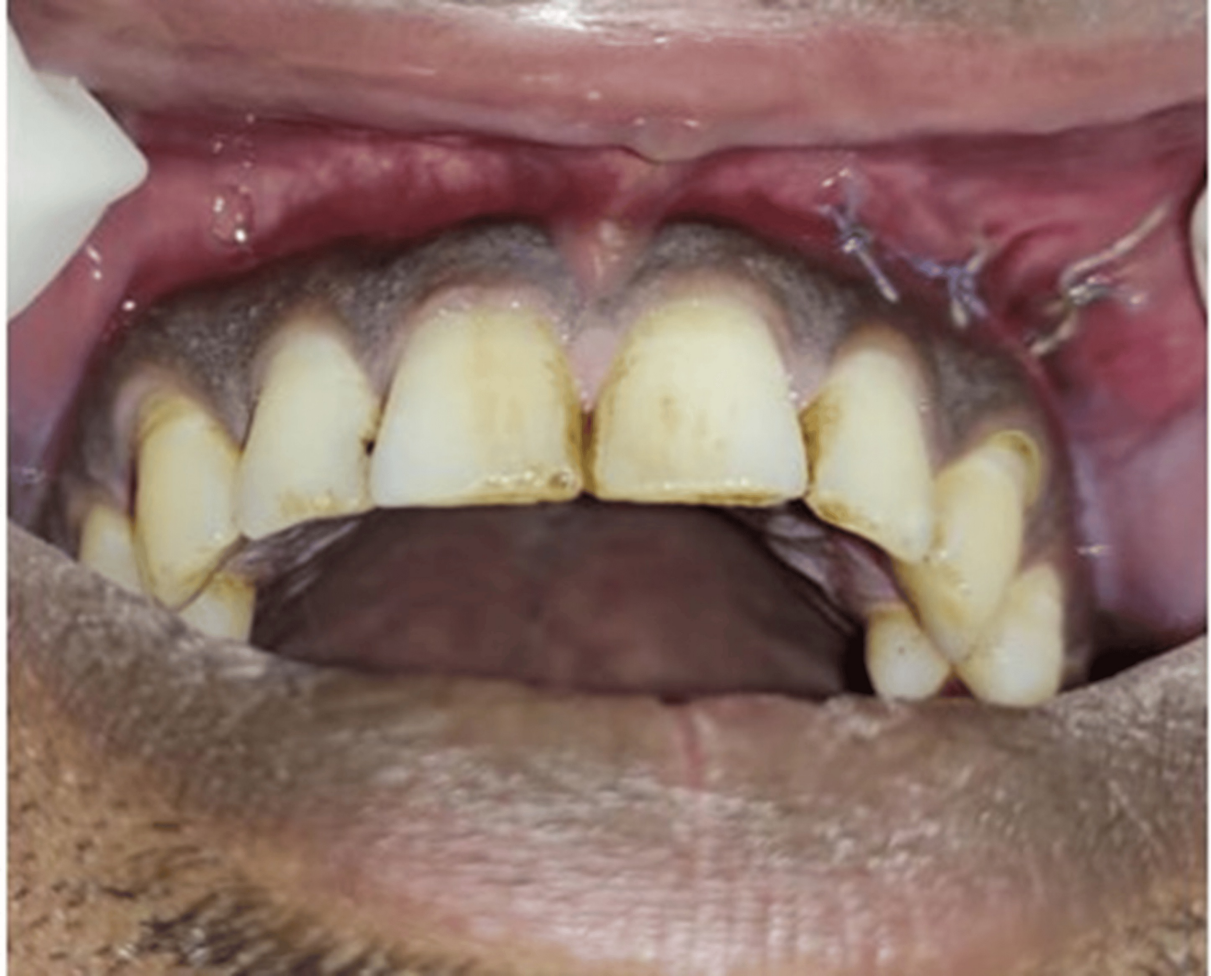
Satisfactory healing of the intraoral surgical site

The patient experienced uneventful healing at the surgical site (Figure 5).

**Figure 6:**
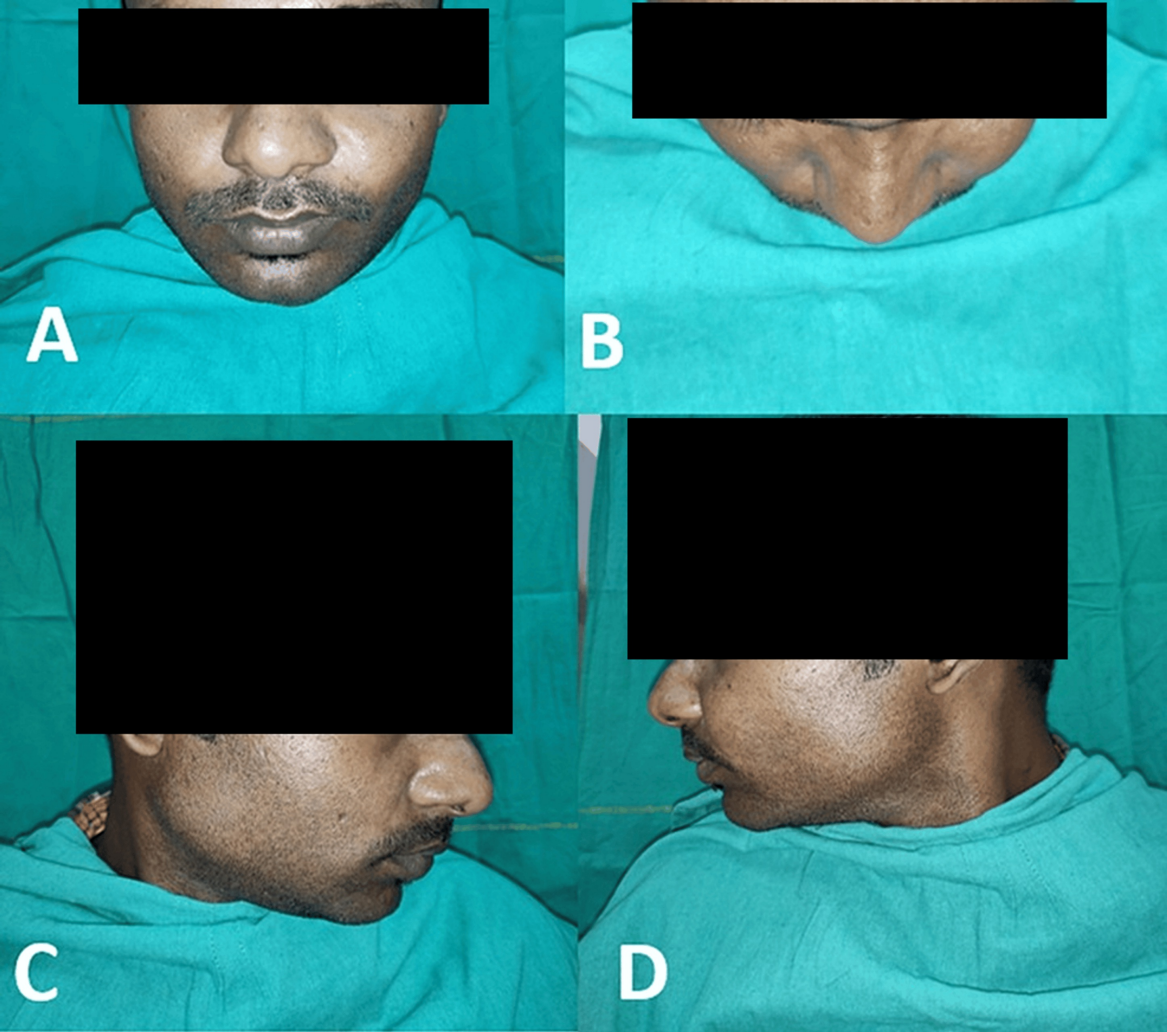
Resolution of extraoral swelling

He is currently under a two-year follow-up and has experienced complete resolution of pain and swelling (Figure 6).

## Discussion

The proposed mechanism for POMC formation involves the entrapment of the sinus or nasal mucosa in a wound, as suggested by Kubo [1-6,8]. Anatomically, the Schneiderian membrane of the maxillary sinus consists of a bilaminar structure: a thin layer of pseudostratified ciliated epithelium on the cavernous side and an overlaid periosteum on the osseous side [9]. POMC, a type of implantation cyst, forms when this epithelium becomes trapped along the line of surgical entry. An inflammatory response then stimulates epithelial proliferation and cystic degeneration, which is followed by expansion due to the osmotic pressure difference from surrounding tissues, resulting in a true cystic cavity anatomically separated from the sinus [10]. Potential causes include maxillary osteotomies, alveolar bone grafting, sinus lift procedures, Caldwell-Luc surgeries, or even traumatic maxillary tooth extractions [1,2]. In rare cases, POMC has been reported in the mandible, likely due to contamination during bi-jaw surgeries with instruments or graft materials containing attached respiratory epithelium [5].

During the Second World War and for several years afterward, POMCs were common in Japan, although they were rare in the Western world. This prevalence is attributed to the surgical treatment of sinusitis during a period when antibiotics were scarce, with POMCs accounting for approximately 20% of oral cysts in Japan [2,3,8,10,11].

POMC may present anywhere from five months to 56 years after trauma or surgery [1,6]. Often, there is a prolonged latency period between the inciting event and the diagnosis [1,2,4,5]. In this case, the patient experienced a traumatic tooth extraction eight months prior and an enucleation five months prior, making it challenging to determine which intervention contributed to the formation of the POMC.

POMC typically occurs in individuals aged 21-72 years, with a higher incidence in the fourth to sixth decades of life. There is a slight male predilection, with a male-to-female ratio of 1.25:1 [1,2,6]. Clinically, POMC presents with pain and/or swelling in the buccal region, often involving adjacent teeth and causing maxillary discomfort. Secondary infections may lead to pus discharge. Radiographically, it appears as a corticated unilocular radiolucency, often with cortical perforation [4].

When associated with nonvital teeth, POMC can mimic radicular cysts in both radiological and operative findings. Differentiation relies on the history of midface surgery or trauma and histopathological examination. The key histopathological feature of POMC is the presence of respiratory epithelium in the cyst lining. This epithelium may be ciliated, cuboidal, or squamous with mucous cells. As reported by Gardner in 1986, the underlying connective tissue may be fibrotic or cellular, and features such as cholesterol clefts, hemosiderin, foam cells, and foci of calcification may be observed [8].

Management of POMC typically involves Partsch I (marsupialization) or Partsch II (enucleation) procedures. Partsch I is recommended for extensive lesions with bony perforations or very thin walls, but has a higher risk of recurrence [12]. Enucleation, the preferred treatment due to the benign nature of the lesion, can also trigger POMC formation if the respiratory epithelium is incompletely removed or re-implanted during the surgical procedure [4].

To prevent POMC formation, meticulous surgical techniques must be employed. Extraction of maxillary teeth in close proximity to the sinus should be performed atraumatically, with delicate handling of the sinus lining to avoid oroantral communication. Suture placement post-extraction should include only the oral mucosa, avoiding the incorporation of the sinus epithelial lining. Additional measures include suturing torn nasal mucosa, performing mandible surgery first, changing saw blades after maxillary osteotomies, thoroughly irrigating osteotomy cuts and fracture fragments to flush any trapped respiratory epithelium, avoiding residual maxillary or nasal autografts in other sites, and preventing rupture of the Schneiderian membrane during sinus lift procedures [4]. Trans-nasal FESS is a newer treatment modality for POMC but is criticized for its risk of recurrence [1].

## Conclusions

POMCs are rare sinus pathologies. Various causes can lead to their development, with iatrogenesis being one, as demonstrated by the reported case. Although these cysts are not common, they should still be included in the differential diagnosis of radiolucent lesions in patients with a history of trauma or surgical intervention in the maxillary sinus region. They may also be considered in the differential diagnosis of mandibular cysts if there is a history of simultaneous surgery on both the maxilla and mandible. Surgeons should be aware of the potential for iatrogenic seeding of the antral lining during procedures that breach the epithelial lining of the antrum. It is crucial to perform such procedures meticulously to avoid complications, such as entrapment of the respiratory epithelium during suturing. When possible, the Caldwell-Luc approach should be avoided, with transnasal endoscopic access considered to prevent infiltration of the sinus mucous membrane and further implantation of respiratory epithelium in the maxilla or mandible.

Lack of awareness about this pathological entity often leads to misdiagnosis as inflammatory cysts of dental origin, resulting in delayed management. Accurate diagnosis hinges on a comprehensive patient history that identifies any breach in the sinus lining, whether surgical or traumatic, and histopathologic evidence of respiratory epithelium in the cystic lining. Given their potential for local destruction, prompt diagnosis followed by enucleation of the lesion - ensuring complete removal of the cystic lining and taking precautions to prevent recurrence and minimize morbidity - is the optimal management approach.
